# coda4microbiome: compositional data analysis for microbiome cross-sectional and longitudinal studies

**DOI:** 10.1186/s12859-023-05205-3

**Published:** 2023-03-06

**Authors:** M. Luz Calle, Meritxell Pujolassos, Antoni Susin

**Affiliations:** 1 Biosciences Department, Faculty of Sciences, Technology and Engineering, University of Vic - Central University of Catalonia, Carrer de La Laura, 13, 08500 Vic, Spain; 2 Mathematical Department, UPC-Barcelona Tech, Barcelona, Spain

**Keywords:** Compositional data analysis, Log-ratio analysis, Longitudinal studies, Microbiome analysis, Microbial signatures, Penalized regression

## Abstract

**Background:**

One of the main challenges of microbiome analysis is its compositional nature that if ignored can lead to spurious results. Addressing the compositional structure of microbiome data is particularly critical in longitudinal studies where abundances measured at different times can correspond to different sub-compositions.

**Results:**

We developed *coda4microbiome*, a new R package for analyzing microbiome data within the Compositional Data Analysis (CoDA) framework in both, cross-sectional and longitudinal studies. The aim of *coda4microbiome* is prediction, more specifically, the method is designed to identify a model (microbial signature) containing the minimum number of features with the maximum predictive power. The algorithm relies on the analysis of log-ratios between pairs of components and variable selection is addressed through penalized regression on the “all-pairs log-ratio model”, the model containing all possible pairwise log-ratios. For longitudinal data, the algorithm infers dynamic microbial signatures by performing penalized regression over the summary of the log-ratio trajectories (the area under these trajectories). In both, cross-sectional and longitudinal studies, the inferred microbial signature is expressed as the (weighted) balance between two groups of taxa, those that contribute positively to the microbial signature and those that contribute negatively. The package provides several graphical representations that facilitate the interpretation of the analysis and the identified microbial signatures. We illustrate the new method with data from a Crohn's disease study (cross-sectional data) and on the developing microbiome of infants (longitudinal data).

**Conclusions:**

*coda4microbiome* is a new algorithm for identification of microbial signatures in both, cross-sectional and longitudinal studies. The algorithm is implemented as an R package that is available at CRAN (https://cran.r-project.org/web/packages/coda4microbiome/) and is accompanied with a vignette with a detailed description of the functions. The website of the project contains several tutorials: https://malucalle.github.io/coda4microbiome/

**Supplementary Information:**

The online version contains supplementary material available at 10.1186/s12859-023-05205-3.

## Background

Although there are still many unknowns about the specific mechanisms of action of the human microbiome, there is growing evidence of its relevance in human health [24, 38]. In recent years, much progress has been made in microbiome research thanks to high-throughput DNA sequencing technologies that allow precise quantification of the composition of the microbiome. The study of the microbiome is considered a great opportunity for improving the current treatment of some diseases and for deriving microbial biomarkers that could be used as diagnostic or prognostic tools.

Microbiome composition is dynamic and the study of microbiome changes over time is of primary importance for understanding the relationship between microbiome and human phenotypes. Longitudinal studies are costly, both economically and logistically, but there is growing evidence of the limitations of cross-sectional studies for providing a full picture of the role of the microbime in human health. Microbiome longitudinal studies can be very valuable in this context, provided appropriate methods of analysis are used [34]. The analysis of microbiome data involves significant experimental and computational challenges [5]. One of them is the compositional nature of the data, which requires the use of specific methods of analysis [9, 17–19]. Compositional data refers to constraint multivariate non-negative data that carry relative information. Microbiome relative abundances (proportions) are constrained by a total sum equal to one. This total constraint induces strong dependencies among the observed abundances of the different taxa. In fact, the observed abundance of each taxon is not informative and only provide a relative measure of abundance when compared to the abundances of other taxa [36]. Ignoring the compositional nature of microbiome data can lead to spurious results [28, 37]. This is particularly critical in the context of microbiome longitudinal studies where compositions measured at different times can be affected by distinct batch effects and similar quality control or filtering protocols may yield to different sub-compositions at each time point.

Aitchison [2] laid the foundations of Compositional Data Analysis (CoDA), which relies on extracting the relative information of compositional data by comparing the parts of the composition. Logarithms of ratios between components (log-ratios) are the fundamental transformation in this framework [20, 31] and is known as the log-ratio approach.

Some methods used in microbiome analysis, such as *ALDEx2* [13], *LinDA* [39, 40], *ANCOM* [26], *ANCOM-BC* [23], *fastANCOM* [39] and *LOOCM* [21], perform the log-ratio approach to identify differential abundant taxa between two study groups. Here we introduce *coda4microbiome*, a new R package for analyzing microbiome data within the CoDA framework in both, cross-sectional and longitudinal studies. *coda4microbiome* is an improvement of our previous algorithm, *selbal* [33], using a more flexible model and a more computationally efficient global variable selection method that results in a considerable reduction of computational time. *coda4microbiome* differs from most differential abundance (DA) testing methods that aim to characterize microbial communities by selecting taxa with significant different abundances between two study groups (*e.g.*, controls vs cases). Like *selbal*, the aim of *coda4microbiome* is prediction, *i.e.*, the method is designed to identify a model (microbial signature) containing the minimum number of features with the maximum predictive power. The algorithm relies on the analysis of log-ratios between pairs of components and variable selection is addressed through penalized regression on the “all-pairs log-ratio model”, the model containing all possible pairwise log-ratios.

For longitudinal data, pairwise log-ratios measured at different time points gives a curve profile or trajectory for each sample. A summary of the shape of these individual trajectories will be the basis for the analysis. More specifically, the algorithm infers dynamic microbial signatures by performing penalized regression over the summary of the log-ratio trajectories (the area under these trajectories).

In both, cross-sectional and longitudinal studies, after reparameterization of the initial “all-pairs log-ratio model”, the inferred microbial signature is expressed as a function of the (log-transformed) initial variables in the form of a log-contrast model [3], *i.e.*, a log-linear model with the constraint that the sum of the coefficients is equal to zero. The zero-sum constraint ensures the invariance principle required for compositional data analysis. These microbial signatures can be interpreted as the (weighted) balance between two groups of taxa, those that contribute positively to the microbial signature and those that contribute negatively. For longitudinal data and a binary outcome (*e.g.* disease status), the signature provides two groups of taxa with different log-ratio trajectories for cases and controls.

The algorithm is implemented in the R package “code4microbiome” (https://cran.r-project.org/web/packages/coda4microbiome/). Several graphical representations of the results are provided that facilitate the interpretation of the analysis: plot of the log-ratio trajectories, plot of the signature (selected taxa and coefficients) and plot of the prediction accuracy of the model. In fact, *coda4microbiome* is not just an R package but a broader initiative that aims to bridge the gap between compositional data analysis and microbiome research. To this end, we are conducting training activities and developing materials that are available at the website of the project: https://malucalle.github.io/coda4microbiome/

The methodology for cross-sectional data is described in “Microbioal signature based on log-ratio analysis: cross-sectional studies” Section and illustrated with data from a pediatric Crohn's disease study (“Cross-sectional data: Crohn’s disease (CD) study” Section). The methodology for longitudinal data is described in “Microbial signature based on log-ratio analysis: longitudinal studies” Section and illustrated in “Longitudinal data: early childhood and the microbiome study” Section with data from the “Early childhood and the microbiome (ECAM) study” [7, 8]. We performed a simulation study for benchmarking of several microbiome analysis algorithms (“Simulation study” and “Simulation study results” Section) and applied *coda4microbiome* in several real datasets (“Real datasets analysis” Section).

## Materials and methods

### Microbioal signature based on log-ratio analysis: cross-sectional studies

Assume we have *n* subjects with phenotype $Y = \left( {Y_{1} , \ldots ,{ }Y_{n} } \right)$ and denote by $X_{i} = \left( {X_{i1} ,{ }X_{i2} , \ldots ,X_{iK} } \right)$ the microbiome composition of subject *i* for *K* taxa. $X$ can represent either relative abundances (proportions) or raw read counts. We approach the identification of those taxa that are associated to the outcome through penalized regression on the “all-pairs log-ratio model”, a generalized linear model containing all possible pairwise log-ratios [6]:

1 $$g\left( {E\left( Y \right)} \right) = \beta_{0} + \mathop \sum \limits_{1 \le j < k \le K} \beta_{jk} \cdot{\text{log}}\left( {X_{j} /X_{k} } \right)$$

The regression coefficients in Eq. (1) are estimated to minimize a loss function $L\left( \beta \right)$ subject to an elastic-net penalization term on the regression coefficients$:$

2 $$\hat{\beta } = \mathop {{\text{argmin}}}\limits_{\beta } \left\{ {L\left( \beta \right) + \lambda_{1} ||\beta||_{2}^{2} + \lambda_{2} ||\beta||_{1} } \right\}$$

A common reparameterization of the penalization parameters is $\lambda_{1} = \lambda \left( {1 - \alpha } \right)/2$ and $\lambda_{2} = \lambda \alpha$ where $\lambda$ controls the amount of penalization and $\alpha$ the mixing between the two norms.

For the linear regression model the loss function is given by the residual sum of squares

$$\hat{\beta } = \mathop {{\text{argmin}}}\limits_{\beta } \left\{ {Y - M\beta_{2}^{2} + \lambda_{1} ||\beta||_{2}^{2} + \lambda_{2} ||\beta||_{1} } \right\},$$

where $M$ is the matrix of all pairwise log-ratios and has dimension $n$ by $K\left( {K - 1} \right)/2$. The expression of the optimization problem (2) for other models, like the logistic regression, can be found in Friedman et al. [14]. We use the function cv.glmnet() from the R package *glmnet* [14] to solve (2) within a cross-validation process that provides the optimal value of $\lambda$ with a default value for $\alpha$ equal to 0.9. Non-compositional covariates are previously modeled with *Y* and the fitted values are considered as “offset” in the penalized regression.

The result of the penalized optimization provides a set of selected pairs of taxa, those with a non-null estimated coefficient. The linear predictor of the generalized linear model (2) provides a microbial signature score for each individual, $i \in \left\{ {1, \ldots , n} \right\}$, $M_{i} = \mathop \sum \limits_{1 \le j < k \le K } \hat{\beta }_{jk} \cdot{\text{log}}\left( {X_{ij} /X_{ik} } \right)$, which is associated with the phenotype $Y_{i}$. Because of the linearity of the logarithm, the microbial signature *M* can be rewritten in terms of the selected single taxa which is more interpretable than in terms of pairs of taxa:

3 $$M = \mathop \sum \limits_{1 \le j < k \le K } \hat{\beta }_{jk} \cdot {\text{log}}\left( {X_{j} /X_{k} } \right) = \mathop \sum \limits_{j = 1}^{K} \hat{\theta }_{j} \cdot {\text{log}}\left( {X_{j} } \right)$$

where $\hat{\theta }_{j} = \mathop \sum \limits_{k = j + 1}^{K} \hat{\beta }_{jk} - \mathop \sum \limits_{k = 1}^{j - 1} \hat{\beta }_{kj}$, that is, the sum of the coefficients $\hat{\beta }$ that correspond to a log-ratio that involves component *j* [6].

It can be proved that $\mathop \sum \limits_{j = 1}^{K} \hat{\theta }_{j} = 0$ and thus, the microbial signature *M* is a log-contrast function involving the selected taxa (those with $\hat{\theta }_{j} \ne 0)$. This ensures the invariance principle required for proper compositional data analysis and it facilitates the interpretation of the microbial signature. Indeed, expression $\mathop \sum \limits_{j = 1}^{K} \hat{\theta }_{j} \cdot{\text{log}}\left( {X_{j} } \right)$ in (3) can be interpreted as a weighted balance between two groups of taxa, $G_{1}$ and $G_{2}$, the taxa with a positive coefficient vs those with a negative coefficient [36].

### Microbial signature based on log-ratio analysis: longitudinal studies

#### Summary of log-ratio trajectories

Assume *n* subjects with fixed phenotype $Y = \left( {Y_{1} ,{ } \ldots ,{ }Y_{n} } \right)$. Subject *i* has been observed in $L_{i} { }$ time points, $\left( {t_{i1} ,{ }t_{i2} ,{ } \ldots ,{ }t_{{iL_{i} }} } \right)$. We denote by $X_{i} \left( {t_{ij} } \right) = \left( {X_{i1} \left( {t_{ij} } \right),{ }X_{i2} \left( {t_{ij} } \right),{ } \ldots ,X_{iK} \left( {t_{ij} } \right)} \right)$ the microbiome composition of subject *i* at time $t_{ij}$, where *K* is the number of taxa which is assumed to be the same for all the individuals and all the time points. $X_{i} \left( {t_{ij} } \right)$ can represent either relative abundances (proportions) or raw counts. We denote by $logX_{i} \left( {t_{ij} } \right)$ the logarithm transformation of microbiome abundances after zero imputation [27]. The log-abundance trajectory of component A for individual *i* is denoted by $logX_{iA} = \left( {logX_{iA} \left( {t_{i1} } \right), logX_{i2A} \left( {t_{i2} } \right), \ldots ,logX_{iA} \left( {t_{{iL_{i} }} } \right)} \right)$ and the log-ratio trajectory between components A and B for individual *i* is given by:

$$logX_{iA} - logX_{iB} = \left( {logX_{iA} \left( {t_{i1} } \right) - logX_{iB} \left( {t_{i1} } \right), logX_{iA} \left( {t_{i2} } \right) - logX_{iB} \left( {t_{i2} } \right), \ldots ,logX_{iA} \left( {t_{{iL_{i} }} } \right) - logX_{iB} \left( {t_{{iL_{i} }} } \right)} \right)$$

We summarize the log-ratio trajectory between components A and B for individual *i* within two time points $l_{1}$ and $l_{2}$ as the integral of the log-ratio trajectory:

4 $$s_{i} \left( {A,B} \right) = \mathop \smallint \limits_{{l_{1} }}^{{l_{2} }} \left( {logX_{iA} \left( t \right) - logX_{iB} \left( t \right)} \right) dt,$$

where the values of the log-ratio for $t \notin \left( {t_{i1} , t_{i2} , \ldots , t_{{iL_{i} }} } \right)$ are linearly interpolated.

We do not take the absolute value in Eq. (4) because the sign of the integral is informative: Positive values of $s_{i} \left( {A,B} \right)$ correspond to trajectories of component A above trajectories of component B, that is, larger relative abundances of A with respect to B, while negative values represent the opposite. Values of $s_{i} \left( {A,B} \right)$ around zero can represent similar abundances between A and B over time or a non-homogeneous trend between A and B within the observed region.

Another advantage of the summary $s_{i} \left( {A,B} \right)$ is computational. Since the integral is linear, $s_{i} \left( {A,B} \right)$ is equal to the difference between the integrals of log-transformed microbiome abundances of taxa A and taxa B:

$$s_{i} \left( {A,B} \right) = \mathop \smallint \limits_{{l_{1} }}^{{l_{2} }} logX_{iA} \left( t \right)dt - \mathop \smallint \limits_{{l_{1} }}^{{l_{2} }} logX_{iB} \left( t \right) dt$$

Thus, the number of integrals to be calculated is of the order of *K*, the number of taxa, instead of $K\left( {K - 1} \right)/2$, the number of pairwise log-ratios.

#### Microbial signature based on log-ratio analysis

To identify those log-ratios that are most associated with the outcome *Y*, we implement glm penalized regression on the log-ratio summaries of all pairs of taxa:

5 $$g\left( {E\left( Y \right)} \right) = \beta_{0} + \mathop \sum \limits_{1 \le j < k \le K} \beta_{jk} \cdot s\left( {j, k} \right)$$

where $s\left( {j,k} \right)$ is the summary of the log-ratio trajectory corresponding to components $X_{j}$ and $X_{k}$.

Equation (5) is identical to Eq. (1) for cross-sectional studies except for the change of the pairwise log-ratios by the summary of the log-ratios trajectories. Thus, the inference and variable selection process is performed similarly with elastic-net penalized regression within a cross-validation process using cv.glmnet() from the R package *glmnet* [14].

For each individual, $i \in \left\{ {1, \ldots , n} \right\}$, the microbial signature score is given by $M_{i} = \mathop \sum \limits_{1 \le j < k \le K } \hat{\beta }_{jk} \cdot s_{i} \left( {j,k} \right)$. Because of the linearity of the integrals used as summaries of the log-ratio trajectories and following the same reparameterization than in Eq. (3), *M* can be rewritten in terms of the selected single taxa which is more interpretable than the selected pairs of components:

6 $$\begin{aligned} M & = \mathop \sum \limits_{1 \le j < k \le K } \hat{\beta }_{jk} \cdot s\left( {j_{1} ,j_{2} } \right) \\ & = \mathop \sum \limits_{1 \le j < k \le K } \hat{\beta }_{jk} \cdot \mathop \smallint \limits_{{l_{1} }}^{{l_{2} }} logX_{j} \left( t \right)dt - \mathop \sum \limits_{1 \le j < k \le K } \hat{\beta }_{jk} \cdot \mathop \smallint \limits_{{l_{1} }}^{{l_{2} }} logX_{k} \left( t \right)dt \\ & = \mathop \sum \limits_{k = 1}^{K} \hat{\theta }_{j} \cdot \mathop \smallint \limits_{{l_{1} }}^{{l_{2} }} logX_{j} \left( t \right)dt \\ & = \mathop \smallint \limits_{{l_{1} }}^{{l_{2} }} \left( {\mathop \sum \limits_{k = 1}^{K} \hat{\theta }_{j} \cdot logX_{j} \left( t \right)} \right)dt \\ \end{aligned}$$

where $$\hat{\theta }_{j} = \mathop \sum \limits_{k = j + 1}^{K} \hat{\beta }_{jk} - \mathop \sum \limits_{k = 1}^{j - 1} \hat{\beta }_{kj}$$.

Since $\mathop \sum \limits_{k = 1}^{K} \hat{\theta }_{k} = 0$, the microbial signature *M* is the integral of the trajectory of a log-contrast function involving the selected taxa (those with $\hat{\theta }_{k} \ne 0)$ and, similarly to the signatures for cross-sectional data, it can be interpreted as a weighted balance between two groups of taxa, $G_{1}$ and $G_{2}$, the taxa with a positive coefficient vs those with a negative coefficient.

### coda4microbiome main functions

The package *coda4microbiome* [10] contains several functions that implement the proposed algorithms. The method for the identification of microbial signatures in cross-sectional studies (“Microbioal signature based on log-ratio analysis: cross-sectional studies” Section) is implemented in function coda_glmnet() and the method for longitudinal data (“Microbial signature based on log-ratio analysis: longitudinal studies” Section) is implemented in function coda_glmnet_longitudinal().

The library also contains additional functions like plot_signature_curves() that provides a plot of the signature trajectories or filter_longitudinal() that filters those individuals and taxa with enough longitudinal information.

The *coda4microbiome* methodology is visually described with a pictogram in the supplementary material (Additional file 1: Fig. S1).

### Simulation study

We performed a case–control simulation study to evaluate the discrimination (or classification) performance and computational burden of *coda4microbiome* in comparison to other methods used for microbiome analysis: *selbal* [33], *ANCOM-BC *[23], *ALDEx2* [13], *DESeq2* [25], *edgeR* [32], *metagenomeSeq* [30], and *LinDA* [40].

Both, *coda4microbiome* and *selbal,* provide a classification model (microbial signature) that defines how the selected taxa are combined. For the other methods that only provide a set of differentially abundant taxa, the classification model was obtained by fitting a logistic regression model containing the DA taxa. Metagenomic simulated data was generated using faecal samples from the “Global Patterns” dataset [11] as template, following the data generation model described by Weiss et al. [37]. This model generates true positives taxa so that their relative abundances match their real abundance in the environment. As in Weiss et al. [37], some of the simulation parameters were fixed for all scenarios: the number of most prevalent taxa to keep in simulation template (2000 taxa), the number of true positive taxa (100 taxa), and the sequencing depth (2000 reads). Both categories had the same number of samples, being 50 or 100 samples in each group. The effect size of the true positive taxa was set to 1.25, 1.5, 2, 5, 10 or 20. This results in a total of 12 simulated scenarios, and we considered 10 replicates for each one. After simulated metagenomic datasets were generated, taxa with less than 5% of prevalence among samples were removed, and 1 count was added to all taxa abundances to overcome problems with log-transformations.

To evaluate the discrimination accuracy of the different methods, a five-fold cross-validation process was applied. Samples in each simulation set were randomly grouped into five different cv-fold groups, ensuring the same number of cases and controls in each one. For every cross-validation fold, the train set includes all cv-fold groups except one, used for testing the model afterwards. The same cv-fold groups assignment in a simulation set were used for testing all the algorithms. For DA methods, a taxa selection step was performed on the train set based on the significance of the Benjamini–Hochberg adjusted p-value [4] with a threshold of 0.05. Relative abundances of selected taxa were used to fit a logistic regression model able to classify the two groups. *coda4microbiome* and *selbal* were trained on the train set and the obtained microbial signature was evaluated on the test set. For all methods, the measure of performance was the Area Under the ROC Curve (AUC). We also compared the number of taxa selected by each method and the computational time.

## Results

### Cross-sectional data: Crohn’s disease (CD) study

We illustrate *coda4microbiome* algorithm for cross-sectional studies with data from a pediatric Crohn’s disease (CD) study [16]. The dataset, available at *coda4microbiome* package, includes microbiome compositions of 975 individuals, 662 with CD and 313 without any symptoms. The abundance table agglomerated at the genus level contains 48 genera.

**Fig. 1:**
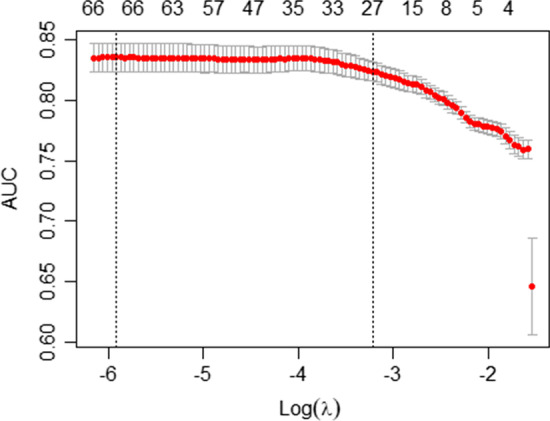
Cross-validation accuracy curve for different degrees of penalization: Log-transformed penalization parameter (x axis), cross-validation AUC (y axis), and, on top of the plot, the number of selected variables for each penalization value. Highlighted with a vertical line the values of "lambda.min" and "lambda.1se" (default penalization value)

We implemented coda4microbiome::coda_glmnet() function to the Crohn’s dataset. The algorithm identifies that the outcome is binary and implements a penalized logistic regression. The results of the analysis provide a first plot (Fig. 1) showing the cross-validation accuracy (AUC) curve from cv.glmnet(). For the default lambda (“lambda.1se”), the algorithm selects 27 pairwise log-ratios that, as we will see later, correspond to 24 different taxa.

**Fig. 2:**
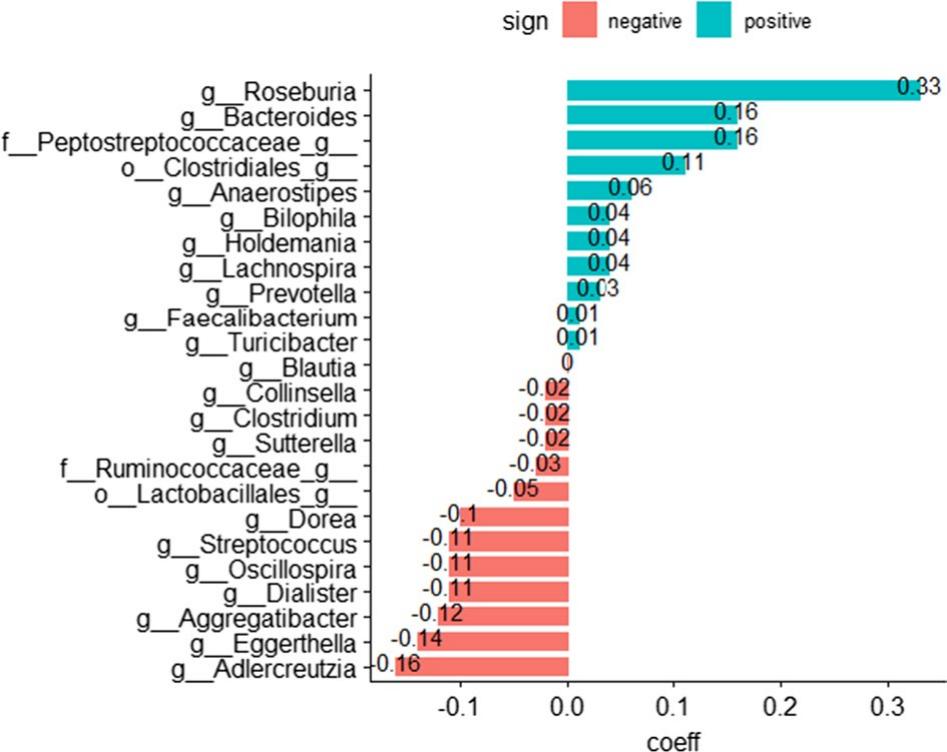
Microbial signature for Crohn’s disease: Taxa composing the microbial signature that best discriminates between Crohn's disease patients and controls. The magnitude of the coefficients represents the contribution of each variable to the model. (green: positive coefficient and red: negative coefficient)

The results of coda_glmnet include the number, the name, and the coefficients of the selected taxa. These can be visualized in a bar plot where the selected taxa and the corresponding coefficients are represented (Fig. 2).

**Fig. 3:**
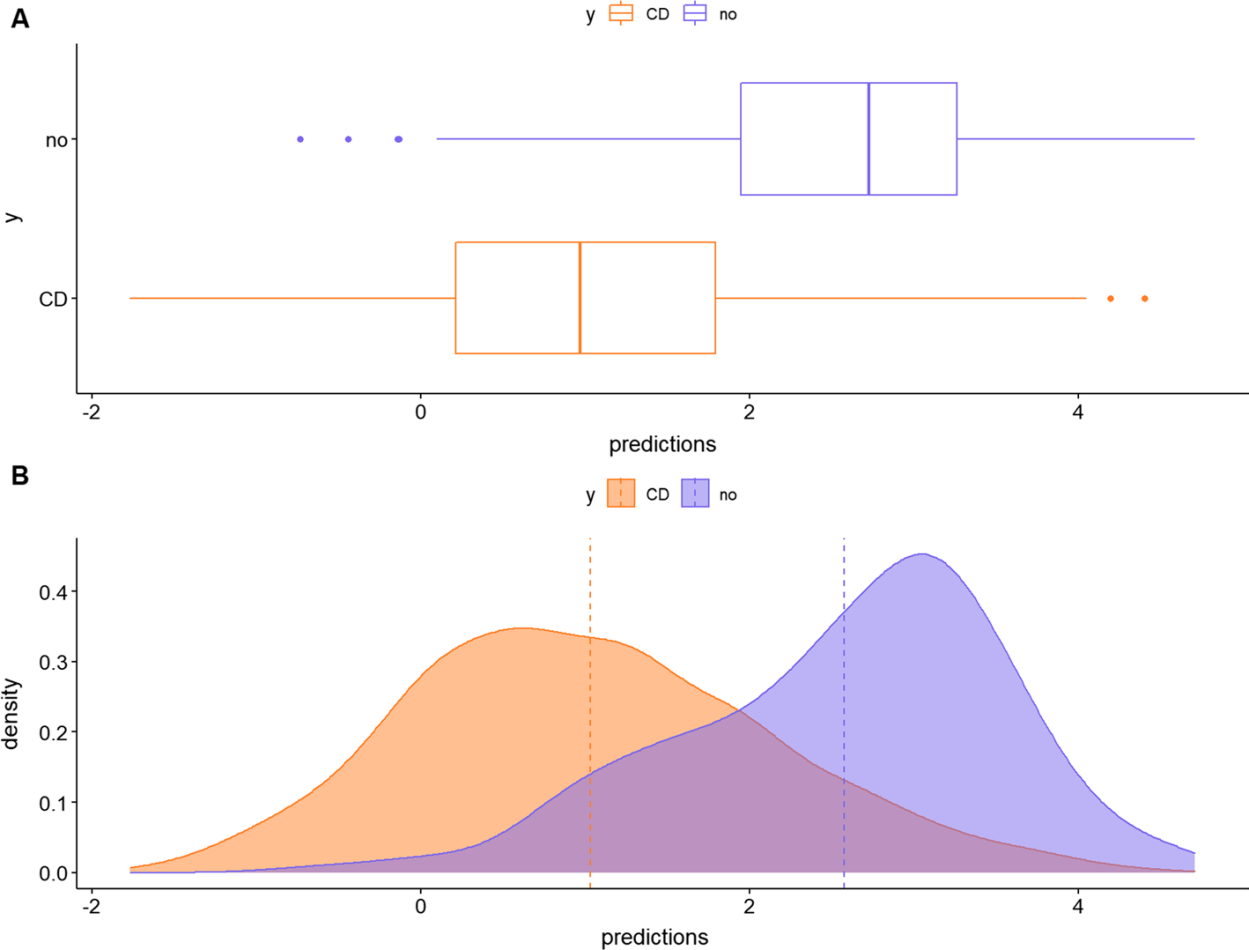
Box-plot and density plots representing the distribution of predicted values (microbial signature scores) for Crohn’s disease patients (orange) and controls (blue)

A third plot describes the discrimination capacity of the selected microbial signature (Fig. 3). This is accompanied with three classification accuracy measures: the apparent AUC, *i.e.*, the AUC of the signature applied to the same data that was used to generate the model, and the mean and sd of the cross-validation AUC obtained from the output of cv.glmnet(). For this dataset, the apparent AUC is 0.84 and the mean (sd) cross-validation AUC are 0.82 (0.0081).

When the outcome is a continuous numerical variable, coda_glmnet() function implements penalized linear regression and Fig. 3 is a scatter plot between predictions and the outcome values.

### Longitudinal data: early childhood and the microbiome study

To illustrate *coda4microbiome* for longitudinal studies we use data from the “Early childhood and the microbiome (ECAM) study” that followed a cohort of 43 U.S. infants during the first 2 years of life for the study of their microbial development and its association with early-life antibiotic exposures, cesarean section, and formula feeding [7, 8].

Metadata and microbiome data were downloaded from https://github.com/caporaso-lab/longitudinal-notebooks. Initially the data contained information on 43 child and 445 taxa at the genus level. We filtered those individuals and taxa with enough information for time-course profiling: we removed individuals with only one time-point observation and those taxa with less than 30 children (70% of individuals) with at least 3 non-zero observations over the follow-up period. After this filtering, the data reduced to 42 children and 37 taxa.

Here we focus on the effects of the diet on the early modulation of the microbiome by comparing microbiome profiles between children with breastmilk diet (bd) vs. formula milk diet (fd) in their first 3 months of life.

**Fig. 4:**
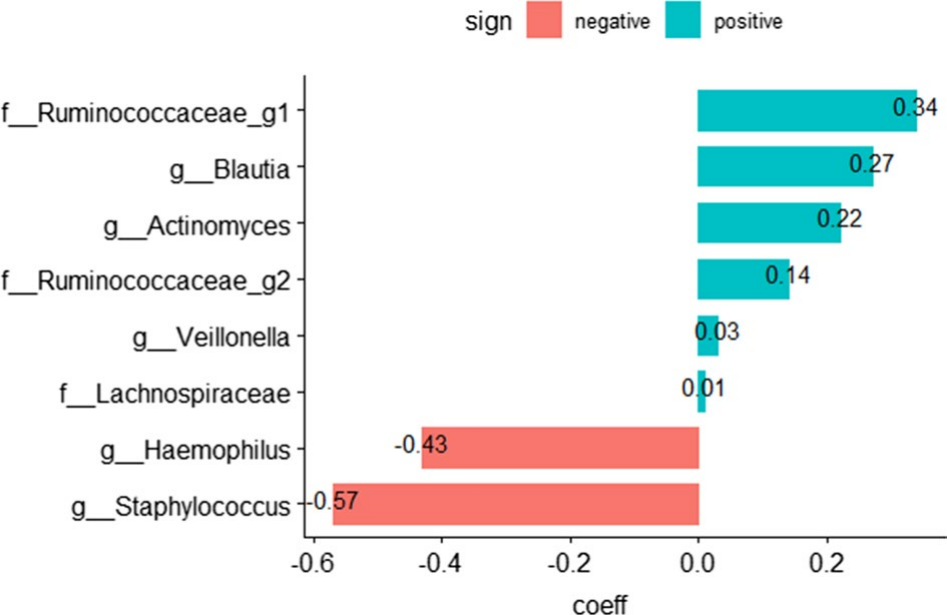
Taxa composing the microbial signature that best discriminates between the two diet groups (green: positive coefficient and red: negative coefficient)

Using function coda_glmnet_longitudinal(), we identified a microbial signature with maximum discrimination accuracy between the two diet groups. The signature is defined by the relative abundances of two groups of taxa, $G_{1}$ and $G_{2}$, where $G_{1}$ is composed of 6 taxa (those with a positive coefficient in the regression model) and $G_{2}$ is composed of 2 taxa (those with a negative coefficient) (Table 1 and Fig. 4). Group $G_{1}$ is mainly dominated by three taxa within the order *Clostridiales* (family *Ruminococcaceae* (2) and gender *Blautia*) and one taxon within the gender *Actinomyces.* Two taxa (*g_Veillonella* and *f_Lachnospiraceae*) have a coefficient close to zero and will have a very small contribution to the signature. Group $G_{2}$ is composed by two taxa within the genders *Haemophilus* and *Staphylococcus.*

**Table 1:** Taxa included in the microbial signature that best discriminates between the two diet groups

Balance group	Coefficient	Taxanomic assignment
*G*_1_	0.3359	*p_Firmicutes;c_Clostridia;o_Clostridiales;f_Ruminococcaceae;g_1*
	0.2730	*p_Firmicutes;c_Clostridia;o_Clostridiales;f_Lachnospiraceae;g_Blautia*
	0.2159	*p_Actinobacteria;c_Actinobacteria;o_Actinomycetales;f_Actinomycetaceae;g_Actinomyces*
	0.1358	*p_Firmicutes;c_Clostridia;o_Clostridiales;f_Ruminococcaceae;g_2*
	0.0337	*p_Firmicutes;c_Clostridia;o_Clostridiales;f_Veillonellaceae;g_Veillonella*
	0.0055	*p_Firmicutes;c_Clostridia;o_Clostridiales;f_Lachnospiraceae;g_*
*G*_2_	− 0.4327	*p_Proteobacteria;c_Gammaproteobacteria;o_Pasteurellales;f_Pasteurellaceae;g_Haemophilus*
	− 0.5672	*p_Firmicutes;c_Bacilli;o_Bacillales;f_Staphylococcaceae;g_Staphylococcus*

**Fig. 5:**
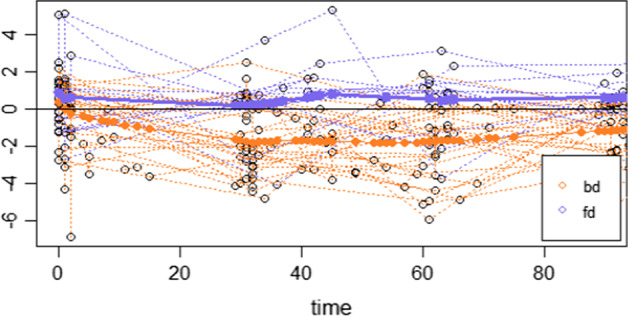
Relative abundance between group $G_{1}$ and $G_{2}$ during the first three months of life. Highlighted curves represent the mean value of the signature for each diet group (orange: breast milk diet, blue: formula milk diet)

The trajectories of the microbial signature over the observed period are represented in Fig. 5, where the color of the curves corresponds to the diet group. Each trajectory represents the relative mean abundances between the two taxa groups for each child. We can see that the two groups are clearly separated. Those children under breastmilk diet (in orange) usually have trajectories below zero, which means they have more relative mean abundance of *g_Haemophilus* and *g_Staphylococcus* with respect to the relative abundance of taxa in group $G_{1}$, while children with formula milk diet (in blue) have more relative abundance of taxa in group $G_{1}$ relative to $G_{2} .$

**Fig. 6:**
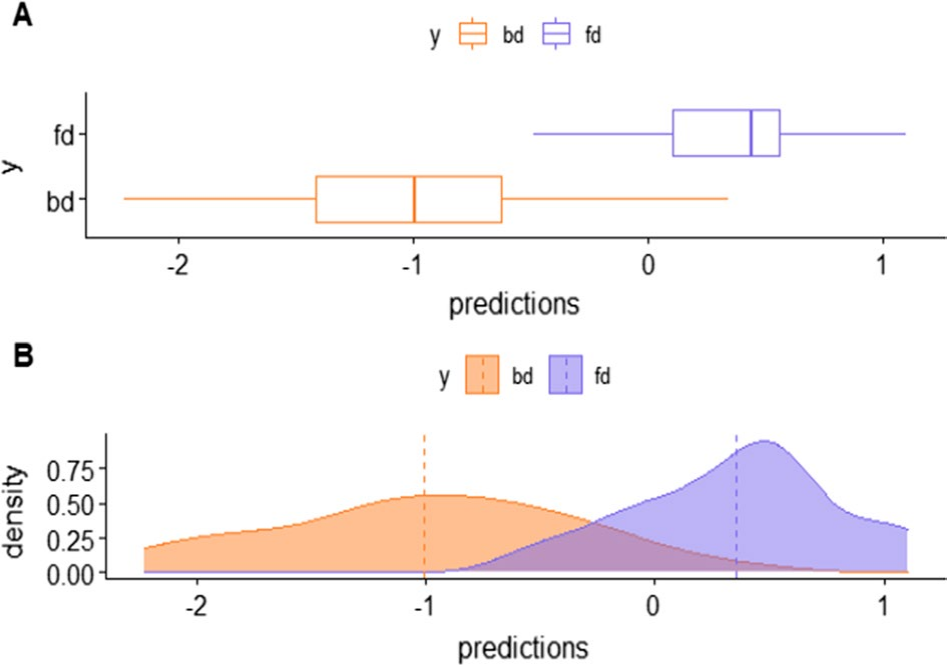
Box-plot and density plots representing the distribution of predicted values (microbial signature scores) for the two diet groups (orange: breast milk diet, blue: formula milk diet)

Figure 6 displays the distribution of the microbial signature scores for the two diet groups and offers a visual assessment of the (apparent) discrimination accuracy of the signature. Quantitatively, the apparent discrimination accuracy of the signature (i. e. the AUC of the signature applied to the same data that was used to generate the model) is 0.96 and the mean cross-validation AUC is 0.74 (sd = 0.10).

The results are consistent with previous studies on the association of the infant gut microbiome composition and breastmilk feeding practices. In Fehr et al. [12], *Haemophilus parainfluenzae* and *Staphylococcus* were found to be enriched with exclusive breastmilk feeding together with lower prevalence of *Actinomyces* at 3 months. *Lachnospiraceae* (*Blautia*) was enriched among infants who were no longer fed breastmilk. Similar results are reported in Laursen et al. [22] where the duration of exclusive breastfeeding was negatively correlated with genera within *Lachnospiraceae* (*e.g.*, *Blautia*) and genera within *Ruminococcaceae*. Positive correlations with exclusive breastfeeding were observed for *g_Bifidobacterium* and *Pasteurellaceae* (*Haemophilus*).

### Simulation study results

**Fig. 7:**
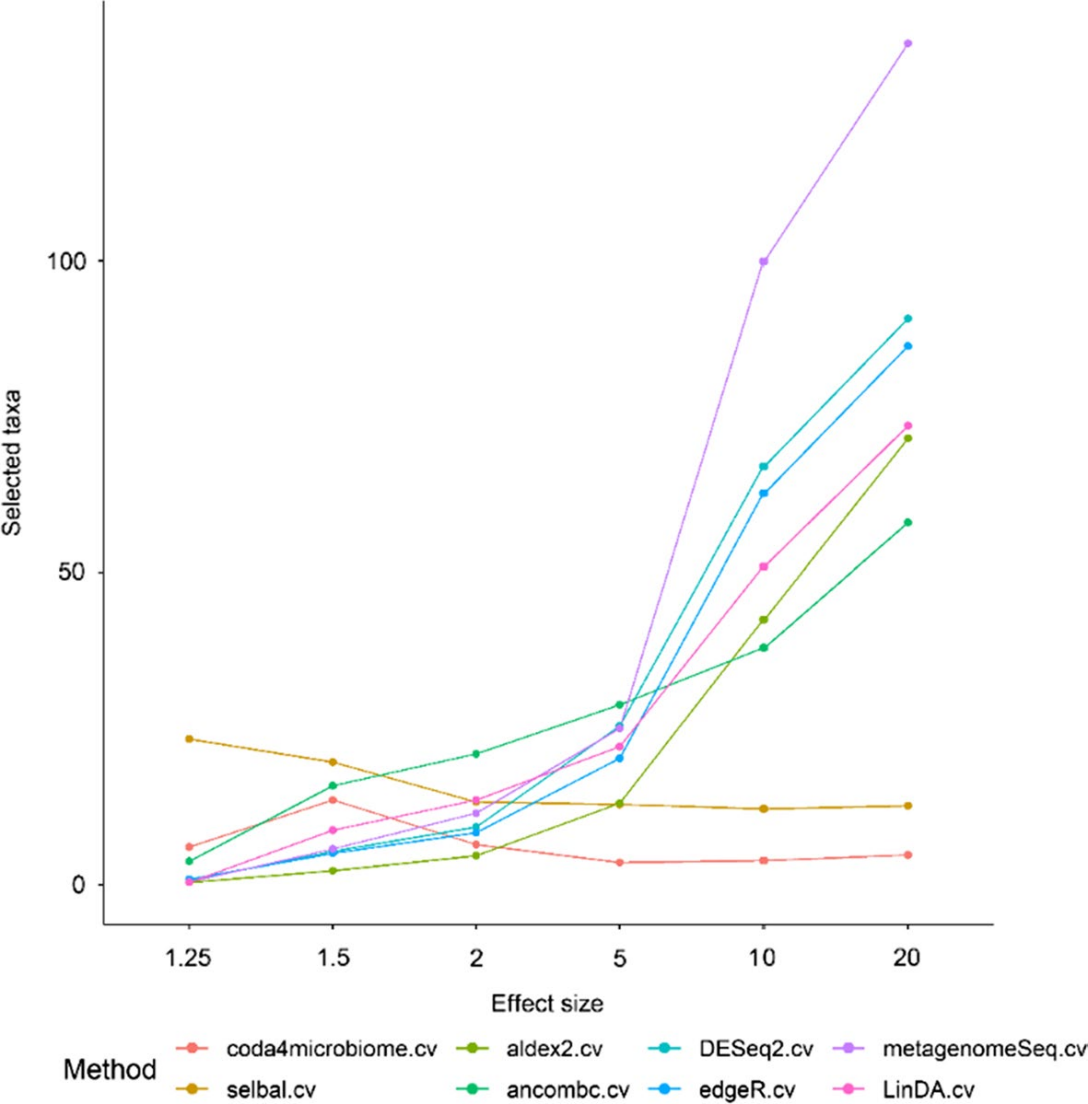
Mean number of selected taxa of the different methods for simulated datasets with different effect sizes (1.25, 1.5, 2, 5, 10 and 20) and 100 samples per group

Figure 7 show the number of selected taxa by each method for simulated datasets with different effect sizes (1.25, 1.5, 2, 5, 10 and 20) and 100 samples per group. Similar results are obtained for simulations with 50 samples per group (results not shown). For all methods, except for *coda4microbiome* and *selbal*, the larger the effect size, the more taxa are selected, as it is expected since the power of the DA tests increases with larger effect sizes. The opposite is true for *coda4microbiome* and *selbal*, as the effect size increases, less variables are needed in the model to obtain good classifications. Despite the fold effect and sample size, *coda4microbiome* finds a predictive microbial signature with less features than *selbal*, with similar AUCs.

**Fig. 8:**
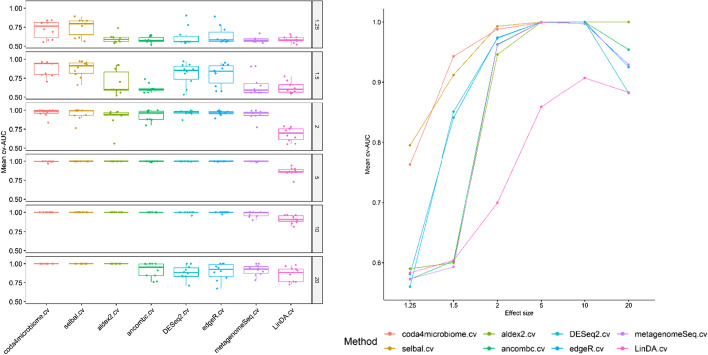
Boxplots distribution and line plots of the mean cross-validation AUCs of every methodology for different effect sizes (1.25, 1.5, 2, 5, 10 and 20) and 50 samples per group

**Fig. 9:**
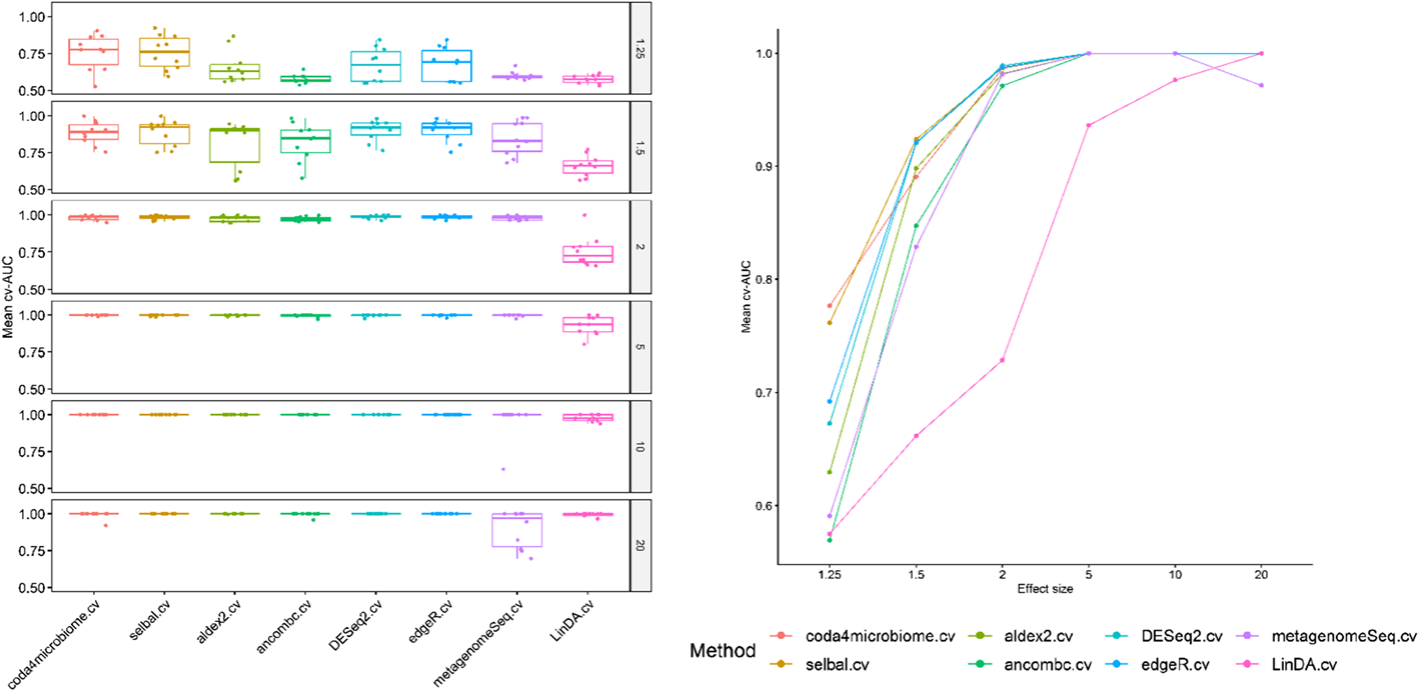
Boxplots distribution and line plots of the mean cross-validation AUCs of every methodology for different effect sizes (1.25, 1.5, 2, 5, 10 and 20) and 100 samples per group

Figures 8 provides two different representations of the discrimination accuracy (AUC) of each method: a boxplot distribution and a line plot of the mean cv-AUC for the 10 replicates of each scenario and 50 samples per group. Figure 9 provides the same information for the case of 100 samples per group. The numerical results (mean and sd) are detailed in Table 2. *coda4microbiome* and *selbal* perform similarly in all scenarios. The higher classification accuracy of *coda4microbiome* and *selbal* is especially remarkable in scenarios with low effect size and a small sample size (Fig. 8). For an effect size equal to 1.25 the mean cv-AUC of these two methods is 0.763 and 0.795, respectively, while all the other methods have mean cv-AUC below 0.6. For an effect size of 1.5, *coda4microbiome* and *selbal* discrimination is 0.943 and 0.912, respectively, and only *DESeq2* and *edgeR* have a good performance, though with lower discrimination values (0.851 and 0.841, respectively). All the other methods methods have mean cv-AUC below 0.6. For larger effect sizes the performance of all the methods if good (discrimination around 1) except for *LinDA* that has a poor performance in all the scenarios. Similar results are obtained for larger sample sizes (Fig. 9). In this case, *DESeq2* and *edgeR* perform very well, with discrimination accuracy still slightly lower than *coda4microbiome* and *selbal* when the effect size is equal to 1.25 but slightly larger to *coda4microbiome* when the effect size is equal to 1.5. All methods, except *LinDA*, reach AUCs over 0.9 in simulations with a fold effect of 2, 5 or 10. On scenarios with very high fold effect, such as 20, classification performance decreases for most of the methods except for *coda4microbiome*, selbal and *ALDEx2*.

**Table 2:** Mean and standard deviation discrimination accuracy (AUC) of the different methods for different effect sizes

Method	n1 = n2 = 50						n1 = n2 = 100					
Effect size	1.25	1.5	2	5	10	20	1.25	1.5	2	5	10	20
coda4microbiome	0.763 (0.12)	0.943 (0.1)	0.988 (0.05)	0.999 (0.01)	1 (0)	1 (0)	0.776 (0.12)	0.891 (0.08)	0.987 (0.02)	1 (0)	1 (0)	1 (0.03)
selbal	0.795 (0.12)	0.912 (0.11)	0.993 (0.07)	1 (0)	1 (0)	1 (0)	0.761 (0.11)	0.924 (0.09)	0.987 (0.01)	1 (0.01)	1 (0)	1 (0)
aldex2	0.59 (0.06)	0.6 (0.16)	0.946 (0.13)	1 (0)	1 (0)	1 (0)	0.629 (0.11)	0.898 (0.16)	0.982 (0.02)	1 (0)	1 (0)	1 (0)
ancombc	0.573 (0.04)	0.603 (0.06)	0.963 (0.07)	1 (0.01)	1 (0)	0.954 (0.1)	0.569 (0.03)	0.847 (0.13)	0.971 (0.02)	1 (0.01)	1 (0)	1 (0.01)
DESeq2	0.56 (0.12)	0.851 (0.15)	0.973 (0.04)	1 (0)	1 (0.01)	0.882 (0.09)	0.672 (0.11)	0.92 (0.07)	0.989 (0.01)	1 (0.01)	1 (0)	1 (0)
edgeR	0.581 (0.12)	0.841 (0.14)	0.974 (0.03)	1 (0)	1 (0.01)	0.925 (0.11)	0.692 (0.11)	0.921 (0.07)	0.988 (0.01)	1 (0.01)	1 (0)	1 (0)
metagenomeSeq	0.573 (0.04)	0.593 (0.14)	0.962 (0.06)	1 (0.01)	0.997 (0.04)	0.929 (0.07)	0.591 (0.03)	0.829 (0.12)	0.982 (0.02)	1 (0.01)	1 (0.12)	0.972 (0.13)
LinDA	0.583 (0.04)	0.604 (0.08)	0.7 (0.09)	0.859 (0.06)	0.907 (0.05)	0.883 (0.1)	0.575 (0.03)	0.662 (0.07)	0.729 (0.1)	0.936 (0.07)	0.976 (0.02)	1 (0.01)

**Fig. 10:**
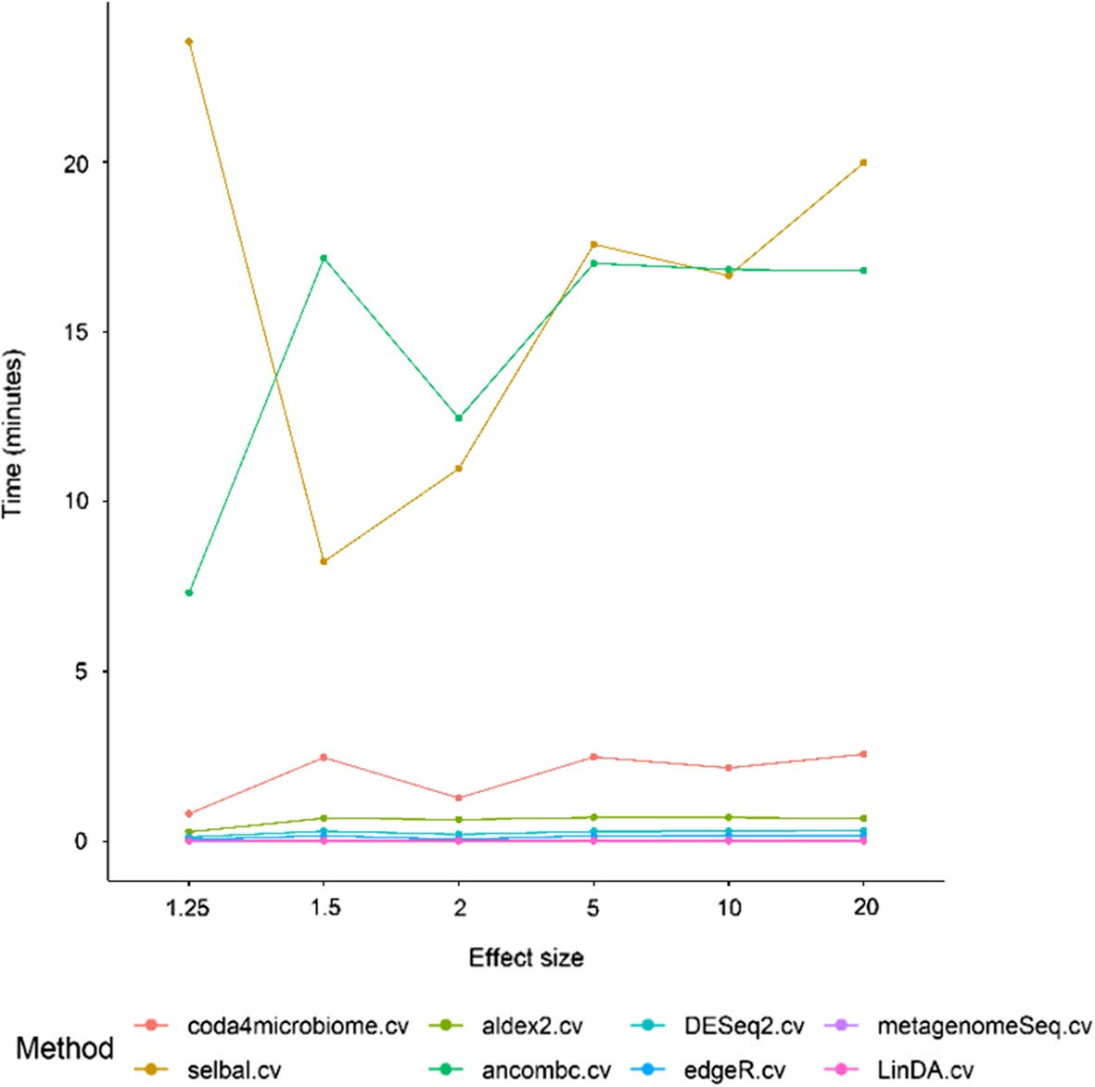
Median computational times for each methodology in simulation datasets with 100 samples per group

Figure 10 show the computational times for each method for different effect sizes (1.25, 1.5, 2, 5, 10 and 20) and 100 samples per group. Similar results are obtained for simulations with 50 samples per group (results not shown). *ANCOM-BC* and *selbal* are the two methods that spent more time in the analysis. *coda4microbiome* is clearly more computationally efficient than *selbal*.

### Real datasets analysis

In order to better compare the computational times of *coda4microbiome* over *selbal*, we applied both methods to 8 real datasets available at [28]. Table 3 shows the great improvement of *coda4microbiome* in comparison with *selbal*, especially when using the recommended function selbal.cv() that, as *coda4microbiome*, implements cross-validation in the analysis and thus, provides more robust results. In fact, because of the computational burden of selbal.cv(), we only could use selbal() function in the simulations. Table 3 is ordered according to the number of taxa in each dataset and this allows to easily see the high correlation between computational time and the number of features.

**Table 3:** Computational times for coda_glmnet() function from R package *coda4microbiome* and functions selbal() and selbal.cv() from R package *selbal*

Dataset	Number of taxa	Total sample size (N1; N2)	coda4microbime coda_glmnet()	selbal()	selbal.cv()
cdi_schubert	75	237 [84;153]	0.057	0.291	7.591
ob_goodrich	117	613 [428;185]	0.112	0.752	18.841
hiv_noguerajulian	140	170 [28;142]	0.071	0.543	14.962
Ji_WTP_DS	155	59 [30;29]	0.088	0.334	9.427
Office	242	625 [341;284]	0.569	2.742	119.696
ArcticFreshwaters	274	1023 [540;483]	0.781	8.156	145.931
Blueberry	418	63 [24;39]	0.942	2.155	68.607
sw_sed_detender	1025	78 [60;18]	27.841	13.76	463.544

## Discussion

*coda4microbiome* algorithm represents an improvement of our previous algorithm *selbal* [33]. Both, *coda4microbiome* and *selbal* search for two groups of taxa, A and B, that are jointly associated with the outcome of interest Y. The main differences between both algorithms are (1) the model for combining the relative abundances of taxa in group A and B, (2) the process for selecting the taxa that will constitute the microbial signature and (3) the type of study that can be approached with each method:

1. *selbal* expresses the microbial signature as an ilr balance between A and B [31], *i.e.*, as the log-ratio of the geometric mean abundances of taxa in group A vs taxa in group B. Instead, *coda4microbiome* microbial signature is expressed as a log-contrast model where those taxa with a positive coefficient define group A, those with a negative coefficient define group B and those with a zero coefficient are not part of the microbial signature.
2. *selbal* performs forward selection and *coda4microbime* implements elastic-net penalized regression variable selection.
3. *selbal* is only available for cross-sectional studies while *coda4microbime* is implemented for both cross-sectional and longitudinal studies.

In summary, *coda4microbiome* improves *selbal* by considering a more general model (an ilr balance is a special log-contrast), a more powerful variable selection process (forward selection does not ensure a global optimum) and can be used in both, cross-sectional and longitudinal studies.

The results of our simulations indicate that when the aim is classification, DA tests followed by fitting a regression model with the selected significant taxa perform worse than *coda4microbiome* or *selbal*, which are methods specifically developed for model prediction. *coda4microbiome* performs very well even in situations where the fold change of the associated taxa is quite low (e.g. 1.25), which is probably the case for most of real microbiome associations. Under such small fold effects, other methods such as *edgeR*, *DESeq2*, *ALEDx2*, *ANCOM-BC*, *MetagenomeSeq* and *LinDA* perform poorly. *Selbal* instead, performs similarly to *coda4microbiome* with good discrimination accuracy for the same simulation scenarios. Though selbal and *coda4microbiome* have similar classification power, the latest requires less computational time which is an important advantage especially for datasets with a large number of features.

## Conclusions

We developed an R package for microbiome analysis that deals with the compositional nature of microbiome data in both, cross-sectional and longitudinal studies. *coda4microbiome* provides a set of functions to explore and study microbiome data within the CoDA framework, with a special focus on identification of microbial signatures that can serve as biomarkers of disease risk and prognostic. The results are expressed as the (weighted) balance between two groups of taxa, those that contribute positively to the microbial signature and those that contribute negatively. The interpretability of results is of major importance in this context. The package provides several graphical representations that facilitate the interpretation of the analysis and the identified microbial signatures.

The main difference between *coda4microbiome* and other CoDA methods that also employ the log-ratio approach, such as *ALDEx2* [13], *ANCOM-BC* [23] or *fastANCOM* [39], is that they perform differential abundance testing while *coda4microbiome* is focused on prediction. *coda4microbiome* improves our previous algorithm, *selbal* [33]. Both have similar performance but coda4microbiome is more computationally efficient.

Longitudinal microbiome studies, especially those focused on the human microbiome, have usually low resolution: the number of individuals is small, each individual has few observation times, the observations of the different individuals are not made at exactly the same time, the data are very variable, the expected behavior of the abundance trajectories is not linear or quadratic, etc. This makes it difficult to justify and implement a parametric modeling of trajectories and limits the use of models for longitudinal data (time series, mixed models). In this context, a description of the trajectories such as the one we propose, although less precise, allows to extract valuable information from the data as we have shown in the example. Other longitudinal data modeling strategies [1, 15, 29, 35] could be used in longitudinal microbiome studies with higher resolution such as laboratory or animal experimental studies. Simulation studies should be performed to assess the performance of *coda4microbiome* for longitudinal microbiome data against other existing methods.

With this new R package, we aim to enhance microbiome analysis by taking into consideration the compositional nature of microbiome data through the use of compositional data analysis methods.

## Supplementary Information

**Additional file 1.** Pictogram of* coda4microbiome* algorithm.

## Data Availability

The algorithm is implemented as an R package *code4microbiome* available at CRAN (https://cran.r-project.org/web/packages/coda4microbiome/) Project name: coda4microbiome. Project home page: https://malucalle.github.io/coda4microbiome/. Operating system(s): Platform independent. Programming language: R. Other requirements: R (≥ 3.5.0). License: MIT + file LICENSE. Any restrictions to use by non-academics: none. The datasets used to illustrate the algorithm is available as a data object in the “coda4microbiome” package.
