# Supplementary material for: coda4microbiome: compositional data analysis for microbiome cross-sectional and longitudinal studies
Source: BMC Bioinformatics. 2023 Mar 6;24:82. doi: 10.1186/s12859-023-05205-3 (PMC9990256; doi:10.1186/s12859-023-05205-3)

coda4microbiome

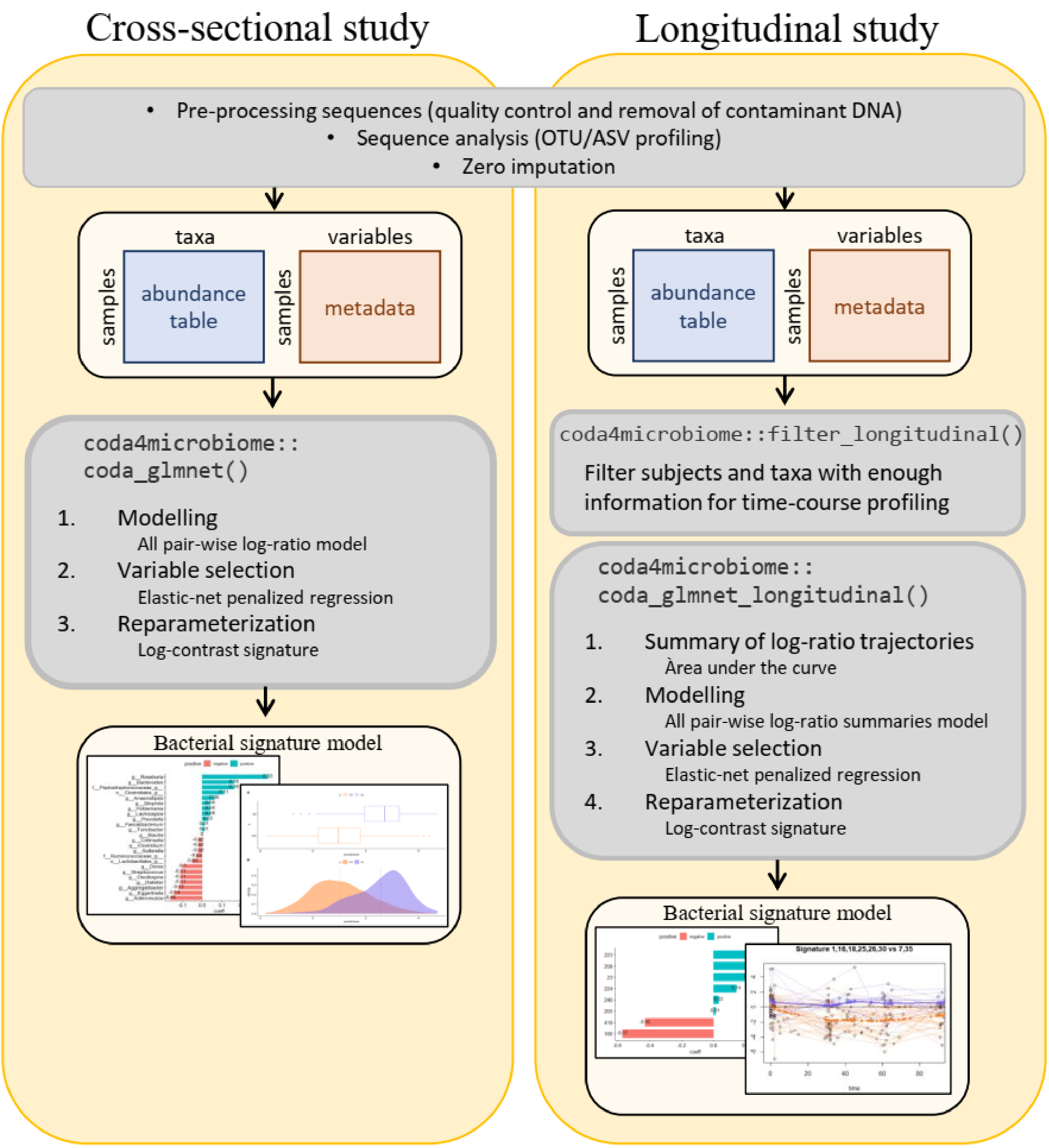

Supplement: Supplementary file 1 — Additional file 1. Pictogram of coda4microbiome algorithm. [file 12859_2023_5205_MOESM1_ESM.pdf]
